# Spatiotemporal Heterogeneity of Macrophages in Acute Pancreatitis: From Inflammatory Initiators to Repair Coordinators and Targeted Therapeutics

**DOI:** 10.1155/mi/3209005

**Published:** 2026-08-02

**Authors:** Xiang Lu, Yu Zeng, Li-hong Gan, Li Li, Jian-qin Liu, Xin Zhou, Xiao-ning Jin, Chao-li Jiang, Zhi Li

**Affiliations:** ^1^ School of Integrated Traditional Chinese and Western Clinical Medicine, Southwest Medical University, Luzhou 646000, Sichuan Province, China, swmu.edu.cn; ^2^ The Key Laboratory of Integrated Traditional Chinese and Western Medicine for Prevention and Treatment of Digestive System Diseases of Luzhou City, The Affiliated Traditional Chinese Medicine Hospital, Southwest Medical University, Luzhou 646000, China, swmu.edu.cn; ^3^ Department of Spleen and Stomach Diseases, The Affiliated Traditional Chinese Medicine Hospital, Southwest Medical University, Luzhou 646000, China, swmu.edu.cn; ^4^ School of Integrated Traditional Chinese and Western Clinical Medicine, North Sichuan Medical College, Nanchong 637000, Sichuan Province, China, nsmc.edu.cn

**Keywords:** acute pancreatitis, macrophages, nanomedicine, single-cell RNA sequencing, spatiotemporal heterogeneity

## Abstract

The current management of acute pancreatitis (AP) primarily relies on supportive measures, such as fluid resuscitation, nutritional support, and infection control. However, these approaches do not adequately address the core drivers of the disease. This limitation arises from an incomplete understanding of the progression from local injury to systemic inflammation and repair, which is a dynamic process with underlying immunoregulatory mechanisms that remain insufficiently characterized. Macrophages are the key effector cells involved throughout the disease course and exhibit pronounced spatiotemporal heterogeneity during this progression. Therefore, they are central to decoding the evolution of the disease and facilitating precise interventions. In terms of spatial dynamics, tissue‐resident macrophages (TRMs), which are derived from embryonic sources, and monocyte‐derived macrophages (MDMs) recruited from the bone marrow serve functionally complementary roles. Their relative dominance shifts in conjunction with disease progression rather than remaining static. Temporally, the macrophage phenotype undergoes a programmed evolution, beginning with an early phase dominated by M1 proinflammatory responses, transitioning through an intermediate phase where injury and repair coexist, and culminating in a late phase characterized by M2‐dominated reparative coordination. This evolution is accompanied by corresponding metabolic reprogramming. Recent single‐cell and spatial multiomics studies have unveiled a functional continuum that goes beyond the traditional M1/M2 dichotomy, revealing a rich diversity of cellular subsets and their spatial niches. This insight shifts targeting strategies from broad anti‐inflammatory interventions toward more precise modulation aimed at specific phases, subsets, and regions. This review systematically examines the design principles, strengths, and limitations of three classes of intervention—molecular targeting, bioactive natural products, and nanoscale delivery—and identifies the obstacles that continue to impede their clinical translation. Building on this analysis, we propose a dual‐dimensional (spatiotemporal) strategy of precise modulation, integrating single‐cell and spatial omics, chronobiological principles, and the traditional Chinese medical concept of *yin shi zhi yi* (adapting treatment to timing), with the aim of shifting AP therapy from symptomatic support toward cause‐directed repair.

## 1. Introduction

Acute pancreatitis (AP) is a prevalent and serious gastrointestinal emergency globally, with its incidence continuing to rise. While most cases are self‐limiting, about 20% progress to severe AP (SAP), which is often accompanied by systemic inflammatory response syndrome (SIRS) and multiple organ dysfunction syndrome (MODS), leading to persistently high mortality rates [1–3]. Current clinical management primarily relies on supportive measures, including fluid resuscitation, nutritional support, and infection control. This approach is fundamentally reactive, addressing pathological outcomes rather than actively intervening against the core drivers of the disease [4]. This limitation highlights our ongoing lack of a systematic understanding of the dynamic progression of AP and its intrinsic immunoregulatory mechanisms, as well as a deficiency in tools for precise modulation.

Recent research has shown that AP is not simply the autodigestion of acinar cells but rather a dysregulated inflammatory cascade driven by innate immunity that rapidly spreads throughout the body [5–7]. In this process, macrophages serve as both key effector cells and regulators of the microenvironment throughout the entire course of the disease [8, 9]. However, the traditional perspective that reduces macrophages to a functionally uniform pro‐ or anti‐inflammatory population is outdated. Macrophages demonstrate significant spatiotemporal heterogeneity in AP. On the spatial level, tissue‐resident macrophages (TRMs) derived from embryonic origins and bone marrow–recruited monocyte‐derived macrophages (MDMs) assume different roles due to their distinct origins, with TRMs favoring homeostatic maintenance and MDMs initiating acute inflammation. On the temporal level, their phenotype evolves dynamically as the disease progresses—from an initial proinflammatory dominance, through a midphase where they engage in both injury and repair, to eventual tissue‐reconstruction dominance.

The heterogeneity of macrophages means that they can participate in protective processes while also potentially aggravating tissue injury. When their spatiotemporal distribution and functional transitions become unbalanced, they can become a critical factor driving disease deterioration. In contrast, targeted and spatiotemporally precise modulation shows promise as a therapeutic strategy. This review is grounded in that central concept, systematically surveying the dynamic evolution and regulatory mechanisms of macrophages in AP. It also critically evaluates the design principles, strengths, and limitations of the corresponding innovative therapies. Based on this analysis, we propose a dual‐dimensional strategy for precise modulation. By integrating the spatiotemporal methods of modern precision medicine with the dynamic, holistic perspective of traditional medicine, this approach aims to shift the paradigm in AP therapy from merely providing symptomatic support to directing causal repair. This direction seeks to offer both a theoretical rationale and a practical path for that transformation.

## 2. Spatiotemporal Heterogeneity of Macrophages in AP

### 2.1. An Integrated Spatiotemporal View of Macrophage Heterogeneity

In AP, macrophages exhibit significant functional differences throughout various stages of the disease and across distinct microanatomical locations within the pancreas. This spatiotemporal heterogeneity is crucial for understanding their dual role. Techniques such as lineage tracing, single‐cell omics, and spatial transcriptomics enable the integration of temporal and spatial dimensions into a cohesive framework. The importance of this framework lies not in merely cataloging the phenotypes at each stage (the essentials of which are summarized in Table 1) but in uncovering three interrelated dynamic relationships.

**Table 1:** Spatiotemporal integrative features of macrophages in acute pancreatitis.

Disease stage	Dominant population (TRM/MDM dynamics)	Polarization tendency/metabolism	Representative single‐cell subset → lineage	Spatial niche	Key signaling axes
Early 0–24 h	Massive MDM influx achieving local dominance; TRMs remain present, providing in situ buffering	M1‐dominant; Warburg	Ly6Chi/IL1B+/S100A9+ → MDM	Around necrotic acini; acinar–stromal interface	CCL2–CCR2; TLR4/NF‐κB; NLRP3; DAMPs such as HMGB1; IL‐10/TGF‐β
Mid 24–72 h	TRMs and MDMs show numerical and functional compensation	M1 still dominant, with internal differentiation already initiated; an M2 shift emerges at 72 h	Arg1+ dual‐phenotype subset, coexpressing Hif1a/Ccr1 and Tgm2/Chil3	Acinar‐to‐ductal metaplasia (ADM) zones; enriched in tissue‐remodeling regions	USP7–PKM2;CSF1R
Late 72 h–several weeks	TRMs reclaim dominance through local proliferation; infiltrating MDMs progressively decline	M2‐dominant; OXPHOS + FAO	Mrc1/Timd4/MerTK+ → predominantly TRM (PDGF–PDGFRα profibrotic type)	Injury–regeneration interface; peristromal/periductal regions	IL‐4/IL‐13–STAT6–PPARγ; PDGF–PDGFRα; TGF‐β; HGF/EGF

*Note:* The time points in this table are based primarily on rodent models; the precise boundaries vary with the induction method and disease severity. The correspondences among the listed subsets, their lineage origins, and spatial niches are largely preliminary mappings based on current functional and transcriptomic evidence and still await joint validation by lineage tracing and spatial omics.

The first relationship is the temporal division of labor among macrophages of different origins. Pancreatic macrophages consist of both embryonically derived, locally self‐renewing TRMs and MDMs [10, 11]. Their relative dominance in AP changes over time rather than reflecting a static division of labor: under homeostasis, TRMs are predominant; within hours to a day following injury, MDMs infiltrate in large numbers via the C–C motif chemokine ligand 2 (CCL2)/C–C chemokine receptor type 2 (CCR2) signaling axis and take local precedence; and as recovery begins, the number of infiltrating MDMs gradually decreases while TRMs, through local proliferation, re‐establish themselves as the dominant population [12, 13].

The second relationship pertains to the connection between single‐cell subsets and their lineage origins. The functional subsets identified through single‐cell sequencing do not correspond one‐to‐one with lineage origins; rather, they illustrate a many‐to‐many relationship, where a single lineage can lead to multiple functional states, and a single functional state may arise from different lineages. Preliminary mappings can be formulated based on existing evidence (see Table 1), yet a key question remains unresolved: The arginase 1 (Arg1^+^) intermediate subset displays transcriptional signatures indicative of both repair‐associated MDMs and plastic TRMs, and its true origin cannot be determined solely from a single‐time‐point expression profile—a distinction that necessitates validation through lineage tracing combined with single‐cell analysis [14, 15].

The third relationship involves the dynamic redistribution of macrophages throughout the disease course. During early inflammation, a significant number of infiltrating MDMs accumulate around injured acini and the surrounding stroma. As the disease progresses, some macrophages become enriched at sites of acinar‐to‐ductal metaplasia (ADM) and tissue remodeling. Upon entering the repair phase, TRMs preferentially expand around the stroma and ducts, driving the remodeling of the extracellular matrix (ECM) [16, 17].

The evidence supporting this spatial distribution is currently uneven in strength. Localization at the stroma–acinus interface is backed by both spatial omics and lineage tracing. In contrast, colocalization within ADM regions and around injured acini relies primarily on functional inference, and robust spatial neighborhood evidence has yet to be provided (see Section 2.5.2).

To synthesize the lineage composition, polarization phenotype, single‐cell subsets, and spatial localization of macrophages across various disease stages within a single framework, the integrated spatiotemporal perspective outlined above is summarized in Table 1. Before examining the dynamic evolution of macrophages in depth, the central conceptual tool adopted here warrants comment: The classical M1/M2 polarization model, as a simplified framework for understanding macrophage functional states, offers a clear frame of reference for organizing the immunological characteristics of the different AP stages. References to “pro‐inflammatory” and “reparative” throughout this article are framed within this model.

### 2.2. The Early Proinflammatory Phase (0–24 h): MDM Influx and M1‐Dominated Inflammatory Initiation

Within hours of injury, the pancreatic immune landscape undergoes rapid changes: MDMs are mobilized from the bone marrow and infiltrate through the CCR2–CCL2 axis [18]. They swiftly adopt an M1 phenotype and activate the NOD‐like receptor family pyrin domain containing 3 (NLRP3) inflammasome, propagating local inflammation systemically. Additionally, they highly express antigen‐processing molecules and secrete Th1‐type cytokines to initiate adaptive immunity (specific markers and signaling pathways are detailed in Table 1) [19, 20]. Spatially, the newly arriving MDMs are situated close to regions of necrotic acini, where they are activated in situ by damage‐associated molecular patterns (DAMPs) such as high‐mobility group box 1 (HMGB1), which are released by acinar cells. This interaction forms an initial site of inflammatory amplification at the acinus–stroma interface [21, 22].

TRMs are present during this stage as well. Using lineage tracing combined with single‐cell RNA sequencing (scRNA‐seq), Baer et al. further resolved pancreatic TRMs into two principal subsets, LYVE1^hi^ and LYVE1^lo^. The LYVE1^hi^ subset is predominantly of embryonic origin, localizes to the stroma, and surrounds ducts and small vessels, exhibiting significant expansion during AP. These cells perform two key functions: They efferocytose apoptotic acinar cells and necrotic debris to disrupt the DAMP cascade while also secreting anti‐inflammatory factors like interleukin‐10 (IL‐10) and transforming growth factor beta (TGF‐β) to counteract excessive nuclear factor kappa B (NF‐κB) activation [23–26]. Thus, the early response is not solely proinflammatory; rather, inflammatory amplification and in situ buffering occur simultaneously and intricately intertwine within the affected area.

The balance between these processes ultimately determines whether the injury remains localized or escalates to systemic dysregulation. This imbalance occurs not only locally in the pancreas but also marks the origin of systemic complications. Simultaneously, M1 activation is evident in distant organs: hepatic Kupffer cells exacerbate systemic inflammation through the release of proinflammatory factors [27], while alveolar macrophages contribute to SAP‐associated acute lung injury [28]. This synchronized, multiorgan inflammatory activation indicates that the early‐phase imbalance acts as the initial trigger in the sequence leading to subsequent systemic complications, including MODS. Furthermore, early M1 polarization is influenced not only by macrophages but also by signals from the microenvironment. Peng et al. [29] demonstrated that the activation of mixed‐lineage kinase domain‐like protein (MLKL) signaling in acinar cells promotes the secretion of CXCL10, which, in turn, enhances macrophage M1 polarization. The knockout of MLKL reduced this effect, while the knockout of receptor‐interacting protein kinase 3 (RIPK3) did not provide comparable protection, suggesting an acinar–macrophage crosstalk mechanism that operates independently of the canonical necroptotic pathway [29].

### 2.3. The Intermediate Transitional Phase (24–72 h): A Window of Both Peak Injury and Repair Onset

This phase represents a crucial turning point in AP pathology, marked by the ongoing progression of injury alongside the simultaneous activation of repair mechanisms. A notable clinical dissociation between serum markers and pancreatic pathology often occurs during this phase. In the pancreatic duct ligation model, serum amylase levels peak at 16 h and subsequently decline rapidly; conversely, the pancreatic histopathology score and levels of proinflammatory cytokines do not reach their peak until 72 h [30]. This dissociation does not indicate that the injury has lessened; rather, the improvement in enzymatic levels reflects a decline in acinar secretory function, as a significant proportion of cells have already died. Meanwhile, the worsening of tissue injury continues to be driven by inflammatory cell infiltration and secondary necrosis.

The pancreatic immune microenvironment undergoes significant changes. Manohar et al. [31] identified several functional subsets among CD206^+^ macrophages by cross‐validating their findings in two independent models. Two subsets, in particular, exhibit notable temporal kinetics: The MHCII^hi^Ly6G^lo^CD44^hi^ subset expands between 24 and 48 h, with a concomitant upregulation of CD54, PDPN, and IL‐22, indicating direct involvement in tissue repair; the MHCII^lo^Ly6G^lo^CD44^hi^ subset shows a peak in LAP–TGF‐β expression at 24 h, returning to baseline by 168 h, suggesting its role in early immune buffering during the peak of injury [31]. Corroborating these findings with the population‐level data from Wu et al. [32] reveals that while M1 macrophages still dominate at 24 h, fine‐grained differentiation has already commenced within the CD206^+^ compartment. By around 72 h, a clear shift toward an M2 phenotype becomes evident, with ADM activity peaking concurrently [32]. At this stage, macrophages continue to release inflammatory mediators while also secreting growth factors to initiate repair, illustrating the functional duality that underpins the disease’s transition from injury to repair.

The intermediate phase is a critical window for numerical and functional compensation between TRMs and MDMs. In the context of CCR2 deficiency, the recruitment of Ly6C^hi^ MDMs is impaired; however, the ADM and repair processes are not significantly affected. This suggests that post‐AP repair does not strictly rely on CCR2‐mediated MDMs. Further research indicates that TRMs, particularly the LYVE1^hi^ subset, depend on colony stimulating factor 1 receptor (CSF1R) signaling to expand following injury and to maintain homeostasis [26]. This observation highlights a dynamic interplay of numerical and functional compensation between TRMs and MDMs within specific limits, though the signals that trigger this compensation and its upper capacity remain to be elucidated. Spatially, a subset of macrophages is enriched in the regions of ADM and remodeling, with changes in function and localization progressing alongside metabolic conversion and subset differentiation [32].

### 2.4. The Late Repair Phase (72 h to Several Weeks): M2‐Dominated Tissue Reconstruction and TRM‐Driven Fibrosis

As the disease enters the recovery phase, macrophages undergo an overall shift from an M1 toward an M2‐like phenotype (markers and signaling are detailed in Table 1). M2 cells rely on mitochondrial oxidative phosphorylation and fatty‐acid β‐oxidation for energy, a metabolic orientation that provides relatively durable energetic stability and equips them for repair tasks lasting days to weeks [33, 34].

The reparative function of macrophages is strongly time‐dependent. Depletion of macrophages before day 3 blocks ADM formation, whereas depletion after day 3 delays the resolution of inflammation—indicating that macrophages serve distinct roles at different stages of the disease [32]. In the L‐arginine model, macrophages first clear necrotic debris and then secrete hepatocyte growth factor (HGF) and epidermal growth factor (EGF), completing the preparatory groundwork and creating conditions for the orderly homing of regenerating cells [35, 36]. Spatially, these reparative macrophages localize primarily to the injury–regeneration interface, where they form a repair zone together with pancreatic stellate cells (PSCs) and regenerating acini.

In the late phase, TRMs once again become the dominant population. Specific depletion of LYVE1^hi^ macrophages markedly reduces TRMs and substantially attenuates the accumulation and activation of platelet‐derived growth factor receptor alpha (PDGFRα^+^) fibroblasts/PSCs, indicating that LYVE1^hi^ TRMs are key drivers of protective fibrosis in AP [26]. Yet, this TRM‐driven fibrosis is double‐edged: while protective in AP, it may be co‐opted to drive pathological progression in the context of chronic pancreatitis and pancreatic cancer [37, 38]. Because the resolution of inflammation and regeneration occurs almost concurrently, once M2‐mediated repair escapes control, it crosses the protective boundary and becomes pathological fibrosis. This has been observed in models with a prolonged disease course, such as pancreatic duct ligation, in which the M2 proportion and the degree of fibrosis rise in tandem [39].

**Figure 1:**
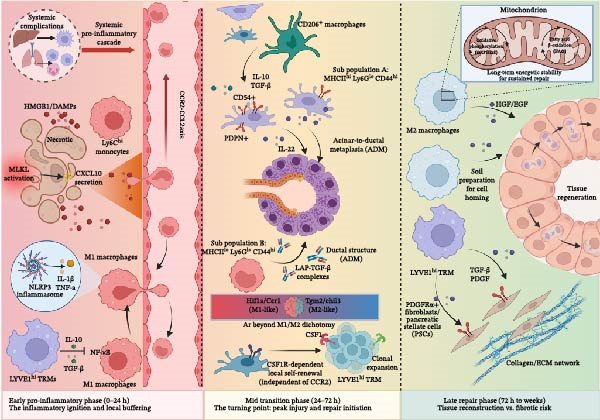
Spatiotemporal heterogeneity of macrophage in acute pancreatitis. *Note:* The diagram illustrates three phases of the disease course—early (0–24 h), middle (24–72 h), and late (72 h to weeks)—highlighting the complementary division of labor between TRMs and MDMs, the M1‐to‐M2 polarization shift, key macrophage subpopulations, and their spatial distribution. It reflects the temporal evolution of inflammation initiation, local buffering, tissue repair, and fibrotic risk.

In summary, macrophages in AP exhibit a programmed temporal evolution: from an early proinflammatory‐dominant phase, through an intermediate transitional phase in which injury and repair coexist, to a late M2‐dominated repair‐dominant phase. This temporal heterogeneity, together with the spatial division of labor between TRMs and MDMs, constitutes the overall picture (Figure 1). The M1/M2 framework, however, is only a simplified point of reference; the true functional spectrum is far more complex—a complexity that the single‐cell and spatial multiomics findings discussed below are now beginning to unravel.

### 2.5. Beyond the Dichotomy: Novel Discoveries From Single‐Cell and Spatial Multiomics

#### 2.5.1. The Single‐Cell Challenge to the Polarization Dichotomy

Single‐cell transcriptomic data are rewriting the traditional polarization dichotomy. The FixNCut study by Aney et al. [40] provides key evidence at the single‐cell level: Arg1^+^ macrophages coexpress M2‐associated genes (Tgm2 and Chil3) alongside proinflammatory genes (Hif1a and Ccr1), indicating that macrophages are not locked into a fixed polarization state but instead undergo continuous dynamic adjustment driven by microenvironmental cues. When combined with the observation from the same study that macrophages return to their preinjury state by day 14, this suggests that such phenotypic continuum is reversible in self‐limiting AP [40].

At the level of population heterogeneity, Fang et al. [41] identified five transcriptomically distinct macrophage subsets in murine AP (Mrc1^hi^, F7^hi^, Htr2b^hi^, Lyz1^hi^, and Gdf3^hi^), a diversity that far exceeds the traditional dichotomy. Meanwhile, Wu et al. [42] constructed the first single‐cell atlas of SAP peripheral blood, identifying a severity‐associated IL1B^+^ CCL3^+^ subset and an SAP‐specific PF4^+^PPBP^+^ subset; CellChat analysis further revealed pronounced activation of signaling axes such as TNF–TNFRSF1B, IL1B–IL1R2, and MIF–CD74.

#### 2.5.2. Spatial Niches and the Spatial Immune–Communication Network

Macrophages are not randomly distributed in the AP pancreas; rather, guided by microenvironmental signals, they form functional aggregates around specific anatomical structures, constituting a spatial immune–communication network. Spatial omics data for human AP, however, are exceedingly scarce, and current understanding derives chiefly from mouse models, with marked differences in evidence strength among niches.

The evidence is most substantial for the stroma–acinus interface: applying spatial transcriptomics to an aging‐ and alcohol‐associated AP mouse model, Tindall et al. [43] found that CD68^+^ macrophages and S100A9^+^ neutrophils infiltrate primarily the pancreatic stromal region, with acinar clusters adjacent to the stroma also showing elevated expression of these markers—suggesting that stromal macrophages may act as intermediaries between acinar injury signals and immune infiltration. This is consistent with lineage‐tracing data from Baer et al. [26] in which LYVE1^hi^ TRMs localize to the stroma, surround ducts and small vessels, and induce the activation of PDGFRα^+^ fibroblasts/PSCs during AP. By contrast, the peri‐injured acinar region and the ADM region are currently based mainly on functional evidence such as immunostaining and single‐cell and flow cytometry time‐series data, while rigorous spatial neighborhood analysis is still lacking.

These niches are not isolated from one another but can be distilled into three interrelated principal axes: a chemotaxis–recruitment axis mediated by CCL2/CXC chemokines, a proinflammatory amplification axis formed by positive feedback loops involving TNF‐α and IL‐1 family members, and a repair–fibrosis axis generated by growth factor networks such as TGF‐β and PDGF [44, 45]. These three axes, however, remain a conceptual synthesis grounded in ligand–receptor expression and functional evidence; their validity as a genuine spatial network awaits future verification of the spatial colocalization and directionality of each axis’s signaling within the niches by integrating spatial omics platforms such as Visium, CosMx, and MERFISH with inference tools such as CellChat and NicheNet [46].

#### 2.5.3. Three Directions of Paradigm Shift

The single‐cell and spatial multiomics findings described above are driving a paradigm shift along three directions: (1) reclassification—borrowing from tumor immunology, subsets are named by their core function or signature markers (e.g., SPP1^+^ matrix–remodeling macrophages and NLRP3^+^ inflammasome–activated macrophages), replacing the simplified M1/M2 labels [47]; (2) translation into diagnostic markers—single‐cell markers such as S100A9, S100A12, and CCL3 hold potential to be translated into circulating biomarkers predictive of SAP severity [42]; (3) precise drug targeting—exemplified by the M1pep–Tasq peptide–drug conjugate, which achieves selective depletion of proinflammatory subsets through a “single‐cell subset identification → marker validation → phage screening → PDC construction” pathway, providing a reproducible paradigm for precision therapy [48].

Single‐cell analysis, lineage tracing, and spatial multiomics together reveal that macrophages in AP constitute a heterogeneous population that is temporally dynamic, spatially precise in localization, and functionally complex. This calls for a shift in therapeutic strategies from “coarse‐grained” anti‐inflammatory intervention toward dual‐dimensional (spatiotemporal) precise modulation directed at specific time points, specific subsets, and specific regions—precisely the core scientific foundation on which the innovative therapies discussed below are built.

## 3. Therapeutic Strategies Targeting Macrophages

**Figure 2:**
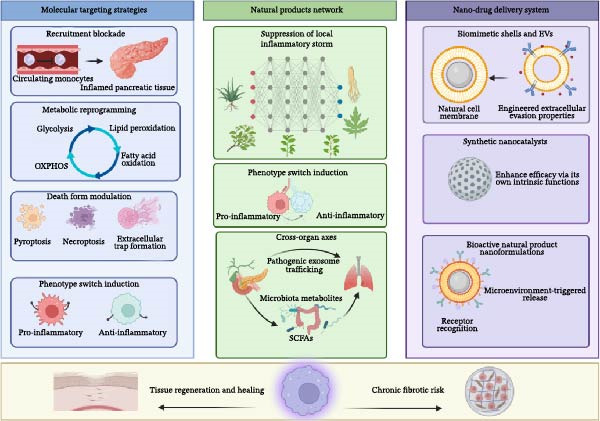
Therapeutic strategies targeting macrophages. *Note*: Current strategies encompass three major domains: molecular targeted therapy, natural products, and nano‐drug delivery systems. Molecular targeting achieves precise intervention by blocking monocyte recruitment, reprogramming metabolism, regulating cell death, and inducing a reparative phenotype switch. Natural products leverage multicomponent networks to suppress the inflammatory storm at the early stage, promote tissue repair at the late stage, and modulate interorgan immune communication (e.g., the gut–pancreas axis). Nano‐drug delivery systems utilize biomimetic cell membrane coating, synthetic nanocatalytic platforms, and microenvironment‐responsive release to achieve spatial enrichment and temporally controlled release.

Advances in understanding the spatiotemporal dynamics and functional heterogeneity of macrophages in AP have driven a shift in therapeutic strategies from broad‐spectrum anti‐inflammatory intervention toward precision modulation. Three classes of strategies can be distinguished by their emphasis: Molecular targeting typically addresses a single node and time point within the dynamic disease process; natural products harness multicomponent networks to orchestrate complex regulation across phases and organs; and nanocarrier‐based delivery exploits microenvironment responsiveness and membrane‐mediated homing to achieve temporally controlled drug release and spatial enrichment. These targeting strategies are summarized in Table 2 and Figure 2.

**Table 2:** Overview of innovative therapeutic strategies targeting macrophages.

Strategy type	Animal model	Representative drug	Core target	Applicable stage	Spatial dimension	Primary effects	Reference	Level of evidence
Molecular‐targeted intervention	TC‐AP	ISO‐1	P38MAPK/NF‐κB	Early	Pancreas and lung	Attenuates pancreatic and pulmonary histopathological injury in AP rats	[49, 50]	Preclinical models + correlative serum‐biomarker evidence
	CER‐AP; PDL‐AP	Prexigebersen	NLRP3/NF‐kB	Early	Pancreas and lung	Attenuates pancreatic and pulmonary histopathological injury in AP rats	[51]	Preclinical models + human clinical trial in another disease
	CER‐AP; FAEE‐SAP	KAN0438757	PFKFB3	Early	Pancreas (local)	Effectively alleviates oxidative stress and inflammation in AP and SAP models	[52, 53]	Preclinical models only
	CER‐AP; FAEE‐AP; NaTC‐SAP	FK866	NAMPT	Early	Pancreas (local)	Inhibits M1 polarization and reduces inflammatory‐cell infiltration	[54]	Validation in multiple animal models + correlative serum‐biomarker evidence
	HFD + CER + LPS‐HLAP	SSO	ATMs/CD36	Early	Pancreas (local)	Alleviates ferroptosis of adipose‐tissue macrophages	[55]	Preclinical models only
	CER + LPS‐SAP	JTE‐013	S1PR2/RhoA/ROCK	Early	Pancreas and intestine	Inhibiting macrophage pyroptosis alleviates pancreatic and intestinal mucosal barrier injury in SAP	[56]	Preclinical models only
	PDL + CER‐SAP	DNase I	IL‐33/ST2	Intermediate–late	Systemic	Modulates CARS and contributes to the shift in immune balance	[57]	Preclinical models + human RCT in another disease
	CER + LPS‐SAP; TC‐SAP	GDF11	TGFβR1/SMAD‐2	Intermediate–late	Systemic	Bidirectionally regulates phenotypic balance, alleviating tissue injury and systemic inflammation	[58]	Preclinical models only
	L‐Arg‐AP	Adropin	PPAR‐γ	Intermediate–late	Pancreas and lung	Promotes macrophage polarization toward the M2 phenotype and attenuates pancreatitis‐associated lung injury	[59]	Preclinical models + limited serum data from AP patients
Bioactive natural product constituents	NaTC‐SAP	Baicalein	HMGB1/TLR4/NLRP3	Early	Pancreas (local)	Blocks DAMPs and inhibits M1 polarization	[60]	Preclinical models + human clinical use in another disease
	CER‐AP	Baicalin	p‐MLKL	Early	Pancreas (local)	Blocks DAMPs and inhibits M1 polarization	[61]	Preclinical models only
	CER‐AP	Paeonol	NLRP3	Early	Pancreas (local)	Blocks DAMPs and inhibits M1 polarization	[62]	Preclinical models + limited clinical use in humans (China)
	CER + LPS‐SAP	Procyanidin	ROS/NLRP3	Early	Pancreas (local)	Alleviates oxidative stress and inflammation and suppresses M1 levels	[63]	Preclinical models + human clinical use in another disease
	CER‐AP	Galangin	NF‐κB/NRF2/HO‐1	Early	Pancreas (local)	Inhibits M1 polarization and enhances antioxidant capacity	[64]	Preclinical models only
	CER‐AP	AKBA (acetyl‐11‐keto‐β‐boswellic acid)	NRF2/HO‐1	Early	Pancreas (local)	Inhibits polarization of M1‐subtype macrophages and oxidative stress	[65]	Preclinical models + human RCT in another disease
	CER + LPS‐SAP	Gastrodin	MAPK/NF‐κB	Early	Pancreas (local)	Attenuates the macrophage inflammatory cascade	[66]	Preclinical model + a marketed drug in human clinical use
	CER + LPS‐SAP; NaTC‐SAP	Kinsenoside	TLR4/STAT1	Early	Pancreas (local)	Reduces macrophage infiltration and alleviates the severity of AP injury	[67]	Preclinical models only
	CER + LPS‐SAP	Tectoridin	JNK and p38 MAPK	Intermediate–late	Pancreas and lung	Promotes polarization of alveolar macrophages toward the M2 phenotype	[68]	Preclinical models only
	NaTC‐SAP	Emodin	PPAR γ‐NF‐κB	Intermediate–late	Pancreas and lung	Inhibits pancreatic exosome secretion and promotes polarization of alveolar macrophages toward the M2 phenotype	[69, 70]	Preclinical model + human RCT of a compound (TCM) formula in AP
	NaTC‐SAP	Emodin	PTEN/PI3K/AKT	Early	Pancreas and lung	Reduces exosomal miR‐21‐3p levels and promotes macrophage polarization toward the M2 phenotype	[71]	Preclinical model + human RCT of a compound (TCM) formula in AP
	CER‐AP	Inulin	SCFAs/HDAC3	Intermediate–late	Gut–pancreas axis	Regulates the M1/M2 polarization balance of macrophages, reducing pancreatic injury and remodeling intestinal homeostasis	[72]	Preclinical model + human RCT in another disease
Nano‐drug delivery system	CER‐AP; L‐Arg‐SAP	Anxa1 mRNA	cGAMP‐cGAS‐STING	Early	Early–intermediate	Restores macrophage efferocytosis and attenuates histopathological injury in AP	[73]	Preclinical models only
	ER‐AP; CDE‐AP	MΦ‐NP (L&K)	Phospholipase A2 (PLA2) inhibition	Early	Pancreas (local)	Neutralizes serum PLA2 activity and improves survival	[74]	Preclinical model + ex vivo validation with limited human serum
	CER + LPS‐SAP	Oc‐M2M@HMnO2–CsA NPs	CCR‐2/SSTR2/ROS	Early–intermediate	Vasculature → pancreas	Three‐stage delivery blocks the inflammatory cascade with temporally programmed (sequential) release	[75]	Preclinical model; ciclosporin A is itself already marketed
	CER + LPS‐SAP	F4/80‐modified ADSC‐EVs	—	Early–intermediate	Pancreas (local)	Inhibits M1 macrophages, enhances antioxidant capacity, and suppresses β‐cell ferroptosis	[76]	Preclinical models only
	NaTC‐SAP	UCMSCs‐PRDX6	ROS/p65/IRF7	Intermediate–late	Multiple organs (lung, kidney, heart)	Promotes macrophage polarization toward the M2 phenotype and suppresses SIRS	[77]	Preclinical models only
	CER‐AR42J‐AP (cell); NaTC‐SAP	MiR‐181a‐5p exosome	ZEB2/RACK1	Intermediate–late	Pancreas (local)	Reduces inflammatory‐cytokine secretion and apoptosis and promotes macrophage polarization toward the M2 phenotype	[78]	Preclinical models only
	CER + LPS‐SAP	Cri‐Pt‐CaFePB	ROS scavenging	Intermediate–late	Pancreas (local)	Promotes macrophage polarization toward the M2 phenotype and enhances antioxidant synergy	[79]	Preclinical models only
	CER + LPS‐SAP	mTWNDs	cGAS‐STING	Early	Pancreas (local)	Scavenges mitochondrial ROS and inhibits M1 polarization, suppressing apoptosis of pancreatic acinar cells (PACs)	[80]	Preclinical models only
	L‐Arg‐SAP	BRSNPs	NF‐κB↓/NRF2/HO‐1↑	Early	Pancreas (local)	Synergistic anti‐inflammatory and antioxidant effects alleviate pancreatitis histopathological injury	[81]	Preclinical models only
	CER + LPS‐SAP	M‐CS‐E‐LNC	CPT1	Intermediate–late	Pancreas, liver, intestine, and lung	Alleviates systemic inflammation in SAP and promotes macrophage polarization toward the M2 phenotype	[82]	Preclinical models only
	CER + LPS‐SAP; CER + LPS‐AR42J‐SAP (cell)	EMO@ZIF‐8/heparin	JNK pathway	Early	Systemic	Restores mitochondrial membrane potential and attenuates oxidative stress and inflammation	[83]	Preclinical models only
	CER‐AP; PDL + CER‐SAP	FePTX@CM NPs	mtROS/Golgi‐stress signaling and ZBP1	Early	Systemic	Codelivery of the two drugs enhances efficacy, alleviating macrophage apoptosis and enzyme hypersecretion	[84]	Preclinical models only
	LPS‐RAW264.7‐SAP (cell); FAEE‐SAP; CAE‐AP	mSe‐PP	AKT/mTOR	Intermediate–late	Systemic	Alleviates oxidative stress and inflammation and restores autophagic flux	[85]	Preclinical models only
	CER + LPS‐AR42J‐SAP (cell); CER + LPS‐SAP	MPBZC	ZIF‐8/ROS	Early	Pancreas (local)	Alleviates oxidative stress and inflammation and restores autophagic flux	[86]	Preclinical models only

*Note:* CER‐AP: caerulein‐induced AP model; FAEE‐AP: fatty acid ethyl ester–induced AP model; NaTC‐AP: retrograde ductal infusion of sodium taurocholate–induced AP model; CER + LPS‐SAP: caerulein plus lipopolysaccharide‐induced severe acute pancreatitis model; PDL + CER‐SAP: pancreatic duct ligation plus caerulein‐induced severe acute pancreatitis model; L‐Arg‐AP: L‐arginine–induced acute pancreatitis model; CDE‐AP: choline‐deficient ethionine–supplemented diet–induced acute pancreatitis model; CER‐AR42J: caerulein‐treated AR42J cell model; CER + LPS‐AR42J: caerulein plus lipopolysaccharide‐treated AR42J cell model; LPS‐RAW264.7: lipopolysaccharide‐treated RAW264.7 macrophage model; HFD + CER + LPS‐HLAP: high‐fat diet + caerulein plus lipopolysaccharide‐induced hyperlipidemic acute pancreatitis model.

### 3.1. Molecular‐Targeted Intervention: Precise Modulation of Macrophage Functional Nodes

Molecular‐targeted therapy aims to achieve precise interruption of the inflammatory cascade in AP by modulating the recruitment, polarization, and functional state of macrophages. Because macrophage function varies dynamically with the disease stage and site of action, an ideal molecular intervention must combine precision in both the temporal window and spatial targeting.

#### 3.1.1. Blocking Pathological Recruitment and Inflammatory Initiation

In early AP, large numbers of circulating monocytes infiltrate the pancreas driven by chemotactic signals and rapidly differentiate into proinflammatory macrophages; blocking this recruitment at its source is therefore a key early intervention strategy. Macrophage migration inhibitory factor (MIF) is central to early inflammatory amplification: It promotes macrophage recruitment to sites of injury by binding to its receptor CD74 and helps sustain a proinflammatory phenotype [87]. The MIF inhibitor ISO‐1 can block this signaling axis, markedly attenuating multiorgan injury in a rat model of SAP, while MIF gene knockout in mice further confirms its causal role in pathogenesis [49, 50]. Clinical studies show that early serum MIF levels are strongly correlated with AP severity and pancreatic necrosis, suggesting that MIF can serve both as a therapeutic target and as a biomarker to guide the timing of early intervention [51].

A recent work has also identified growth factor receptor‐bound protein 2 (Grb2) as a new regulatory node of macrophage activation. Integrated bioinformatics and single‐cell sequencing revealed that Grb2 is enriched mainly in macrophages in AP and is markedly upregulated in the M1 subset; inhibiting Grb2 downregulates the NLRP3 inflammasome and NF‐κB signaling pathways, thereby suppressing M1 polarization [88]. These two targets, however, also raise safety concerns for systemic intervention. MIF is widely distributed across immune and endothelial cells, and Grb2 is an even more ubiquitous signaling adaptor protein; systemic inhibition of either may produce off‐target effects and weaken anti‐infective defenses—a particular danger during the phase of infected necrosis [89].

#### 3.1.2. Targeting Metabolic Reprogramming

Metabolic reprogramming is a core mechanism governing macrophage polarization, and targeting its key nodes can intervene in disease progression at different stages—though the bidirectional effects of metabolites across disease stages warrant close attention.

The key glycolytic enzyme 6‐phosphofructo‐2‐kinase/fructose‐2,6‐bisphosphatase 3 (PFKFB3) is markedly overexpressed in macrophages infiltrating the AP pancreas, and its specific inhibitor KAN0438757 activates the nuclear factor erythroid 2‐related factor 2 (NRF2)/heme oxygenase 1 (HO‐1) signaling axis antioxidant pathway and markedly attenuates inflammation, showing protective effects in both cerulein‐induced AP and fatty acid ethyl ester (FAEE)–induced SAP models [52]. PFKFB3 does not, however, act solely as a proinflammatory factor throughout the disease course: Lactate accumulating in the injured microenvironment can activate repair gene transcription through histone lactylation and drive macrophages toward a reparative phenotype, and PFKFB3‐heterozygous mice—owing to insufficient lactate production—show markedly delayed pancreatic repair [53]. Thus, although inhibiting PFKFB3 in early inflammation is beneficial, extending the intervention into the repair phase may impede regeneration by cutting off lactate‐driven repair signals. Yet, no study has defined the temporal boundary between the two phases that would support clinical decision‐making, and there is a lack of real‐time monitoring biomarkers, leaving no basis for determining when to switch from inhibition to promotion.

A similar tension within the NAD^+^ metabolic network reinforces this point. Inhibitors of nicotinamide phosphoribosyltransferase (NAMPT) can suppress M1 polarization by modulating NAD^+^‐dependent metabolic remodeling, whereas supplementing the substrate nicotinamide mononucleotide (NMN) may produce the opposite, proinflammatory effect [54]. Moreover, in obesity‐related SAP, CD36, which is highly expressed by adipose tissue macrophages (ATMs), can promote lipid peroxidation, drive macrophage ferroptosis, and thereby release DAMPs that aggravate pancreatic injury—suggesting that blocking CD36 or inhibiting ferroptosis may represent a potential therapeutic strategy [55]. Both CD36 and NAMPT, however, participate broadly in lipid uptake and immune recognition; therefore, long‐term blockade carries the risks of metabolic disturbances and impaired cell survival and repair. Furthermore, since existing evidence derives largely from obese or short‐term models, the applicability of these interventions to nonobese SAP and their long‐term safety remains to be verified [90, 91].

#### 3.1.3. Modulating Cell Death Pathways

The way macrophages die also shapes the inflammatory microenvironment, with distinct modes of cell death playing different roles across disease stages. Sphingosine‐1‐phosphate receptor 2 (S1PR2) mediates cytoskeletal reorganization and pyroptosis via the RhoA/ROCK pathway; its antagonist can suppress macrophage pyroptosis, reduce inflammatory cytokine release, and effectively alleviate intestinal mucosal barrier dysfunction secondary to SAP. This strategy is primarily protective in early disease by restraining the burst release of inflammatory mediators, and its effect on intestinal macrophages further broadens the therapeutic potential of this target for modulating the gut–pancreas axis [56]. Pyroptosis, however, also serves a protective function by clearing intracellular pathogens during the phase of infected necrosis, and excessive inhibition therefore carries the risk of impairing anti‐infective defenses [92].

Macrophage extracellular traps (METs) in SAP are not merely a by‐product of cell death but are deeply involved in immune regulation during the mid‐to‐late stages of disease. METs have been shown to regulate the compensatory anti‐inflammatory response syndrome (CARS) via the IL‐33/ST2 signaling axis, driving the transition from SIRS to an immunosuppressed state. DNase I and the peptidylarginine deiminase (PAD) inhibitor Cl‐amidine can attenuate the excessive Th2 activation observed in animal models by disrupting the MET network and reducing IL‐33 release [57]. However, MET‐mediated immunoregulation is stage‐dependent and may exert opposite effects in early SIRS and late CARS, making it extremely difficult to determine the optimal timing for intervention; moreover, Cl‐amidine currently has no clinical indication.

#### 3.1.4. Inducing Reparative Phenotype Conversion

In the late inflammatory phase, actively steering macrophages toward a reparative phenotype and restoring immune homeostasis are key to accelerating healing. Growth differentiation factor 11 (GDF11), by activating the TGF‐βR1/SMAD2 pathway, simultaneously suppresses M1‐associated genes and upregulates M2 markers, effectively alleviating pancreatic tissue injury and systemic inflammation [58]. When employing this strategy, however, caution is warranted: Sustained activation of downstream TGF‐β signaling carries a risk of promoting PSC activation and chronic fibrosis, a concern of particular importance in patients with recurrent pancreatitis. The novel metabolic regulator adropin, in turn, promotes M2 polarization by modulating the phosphorylation of peroxisome proliferator–activated receptor γ (PPARγ), thereby attenuating pancreatitis‐associated lung injury [59]. However, as a core regulator of lipid metabolism, PPARγ activation may pose additional metabolic risks in SAP patients with pre‐existing metabolic disturbances such as hyperlipidemia.

Surveying the four dimensions of recruitment, metabolism, death, and repair, it becomes clear that the precision of molecular targeting is invariably constrained by the spatiotemporal heterogeneity of macrophages. The vast majority of targets correspond only to a single node and a single phase within the dynamic disease process, and once an intervention extends beyond its applicable window, the benefit may reverse into harm.

### 3.2. Bioactive Natural Products: Multitarget Network Modulation of Macrophages

The functional fate of macrophages is jointly shaped by temporal and spatial dimensions, making single‐target drugs difficult to sustain efficacy over the entire disease course. The multicomponent, multipathway nature of natural products is well‐suited to the networked phase‐specific modulation required by this heterogeneity.

#### 3.2.1. Early Phase, Local to the Pancreas: Restraining the M1‐Dominated Inflammatory Storm

In early AP, DAMPs released by injured acinar cells rapidly activate locally recruited monocytes and macrophages, driving a self‐amplifying inflammatory cascade through the TLR4–NF‐κB–NLRP3 axis [93]. At this stage, macrophage signal transduction unfolds stepwise from upstream receptor recognition to downstream effector programs, with multiple intracellular pathways activated in parallel and mutually reinforcing one another. Single‐point inhibition often fails owing to bypass compensation, whereas a multicomponent strategy can exert pressure simultaneously at multiple nodes of the cascade. The contrast between baicalein and baicalin best illustrates this principle. Although the two compounds are chemically related, they act at opposite ends of the same signaling cascade: Baicalein binds HMGB1 upstream to block TLR4 recognition [60], whereas baicalin acts downstream to inhibit the oligomerization of phosphorylated MLKL (p‐MLKL), thereby preventing necroptotic macrophages from releasing a fresh wave of DAMPs [61]. Similarly, paeonol and proanthocyanidins act on the oxidative stress–NLRP3 amplification loop, an intermediate link in the cascade; the key pathway of paeonol has been confirmed by an NLRP3 inhibitor reversal experiment [62, 63]. Galangin and acetyl‐11‐keto‐β‐boswellic acid (AKBA), in turn, concurrently engage two parallel pathways within the same time window, simultaneously suppressing proinflammatory signaling and activating antioxidant defenses to protect acinar cells [64, 65]. In addition, gastrodin and kinsenoside act on p38 MAPK nuclear translocation and TLR4 receptor recognition, respectively, thereby covering additional nodes within this signaling network [66, 67].

The common spatiotemporal window of these interventions is “early phase, local to the pancreas”; once the disease progresses to the stage of infected necrosis, the very same targets—TLR4, HMGB1, and others—assume host‐defense functions, and sustained inhibition at this point may prove counterproductive.

#### 3.2.2. Mid‐to‐Late Phase, Repair Stage: Actively Driving Macrophage Functional Conversion

As acinar injury stabilizes, the functional focus of macrophages must shift from clearance to repair—a transition driven by the systematic remodeling of metabolic programs. At this stage, bioactive natural products no longer act merely as suppressors but also as promoters.

Tectoridin does not simply suppress M1 markers; it also blocks the MAPK cascade by targeting the phosphorylation site of extracellular signal‐regulated kinase 2 (ERK2) while markedly upregulating reparative markers such as CD206 [68]. Because it concurrently inhibits the phosphorylation of JNK and p38 MAPK pathways, its broad‐spectrum activity across the MAPK family renders the phenotypic conversion more stable and less susceptible to reversal via single‐pathway compensation [94]. Emodin, in turn, illustrates how a single compound can serve distinct functions across time and space. In the early phase, it contributes to local suppression of inflammation [95]; later, when AP‐associated acute lung injury (AP‐ALI) develops, it activates PPARγ signaling in alveolar macrophages, promoting the coordinated upregulation of lipid synthesis and fatty acid oxidation. This provides membrane lipids and sustains the secretory machinery required for reparative macrophage function [69, 70]. Such multicontext functionality is difficult to achieve with single‐target chemical drugs, underscoring that the clinical positioning of bioactive natural products should be guided by specific spatiotemporal contexts rather than by broad, static labels.

#### 3.2.3. Cross‐Organ, Cross‐Space: Modulating the Immune‐Communication Network

AP is a systemic disease involving multiorgan crosstalk, and certain natural product monomers can act beyond the local pancreas to modulate macrophage function along spatial dimensions such as the “pancreas–lung axis” and the “gut–pancreas axis.”

Serum exosomes in SAP are enriched in miR‐21‐3p; these are not ordinary metabolic waste but carriers of pathogenic information. Once taken up by alveolar macrophages, they induce M1 polarization and thereby establish a new inflammatory focus in lung tissue not yet directly affected by pancreatic injury. Emodin weakens this “pancreas–lung” pathogenic signaling axis by lowering the miR‐21‐3p content of exosomes [71]. Inulin, by contrast, offers an alternative route of action along the “gut–pancreas axis.” It does not act directly on macrophages; rather, by reshaping the gut microbiota and promoting the production of short‐chain fatty acids (SCFAs) such as butyrate, it achieves epigenetic reprogramming of macrophages systemically through the inhibition of histone deacetylases (HDACs). Two lines of reciprocal evidence—the loss of protection in germ‐free mice and the ability of the HDAC3‐selective inhibitor RGFP966 to reproduce the effect—together support this mechanistic chain [72].

Such cross‐organ modulation also faces formidable challenges: Both the exosomes targeted by emodin and the gut microbiota on which inulin depends are highly heterogeneous among patients, making precise patient stratification, optimal timing of administration, and more refined biomarkers essential for clinical translation.

### 3.3. Nanoscale Drug–Delivery Systems: Precision Strategies Targeting Macrophages

Nanotechnology has opened a new avenue for the precise treatment of AP. Through the rational design of nanocarriers, drugs can be delivered to macrophages in a targeted manner, with prolonged circulation, enhanced bioavailability, and reduced systemic toxicity. An ideal delivery system should not only achieve precise spatial localization but also be capable of temporally controlled release in response to the pathological microenvironment [96, 97].

#### 3.3.1. Biomimetic Membranes and Cell‐Derived Carriers

This class of strategies employs naturally sourced cell membranes or extracellular vesicles (EVs) to equip delivery carriers with intrinsic immune compatibility and inflammatory tropism, thereby enabling efficient macrophage targeting and phenotypic modulation. Its core value lies not only in precise delivery but, more importantly, in its ability to adapt to the functional polarization or phenotypic switch of macrophages over the course of disease, allowing dynamic, adaptive intervention rather than adherence to a single predetermined target.

Cell‐membrane–coated nanoparticles acquire immune evasion capability through coating with native cell membranes and, via microenvironment‐responsive design, achieve temporally controlled release suited to different intervention windows. For the early window, lipid nanoparticles carrying annexin A1 (Anxa1) mRNA act by restoring macrophage efferocytosis and suppressing excessive activation of the cGAS‐STING pathway [73]. In addition, the MΦ‐NP (L&K) composite decoy system, constructed by Zhang et al. [74] using the macrophage membrane and melittin, efficiently captures secretory phospholipase A2 (PLA2) and concurrently loads the inhibitor MJ‐33 within its core, raising survival from 0% to 60% in a highly lethal AP model. As research has advanced, a recently constructed multistage targeting system incorporates three spatial targeting mechanisms: It employs an M2 macrophage membrane to target injured vascular endothelium and evade immune clearance via CD47, uses surface‐modified octreotide to bind somatostatin receptor 2 (SSTR2) and suppress pancreatic enzyme secretion, and dissociates to release cyclosporine A in an acidic, high‐reactive oxygen species (ROS) microenvironment—thereby simultaneously blocking the inflammatory amplification cascade at multiple spatial nodes [75].

EVs, by delivering various endogenous signaling molecules, focus on reprogramming macrophages toward a reparative phenotype [98]: F4/80 antibody–modified EVs derived from adipose‐derived stem cells can precisely target macrophages, suppress M1 polarization, and deliver antioxidant signals that ultimately alleviate ferroptosis in islet β‐cells [76]; umbilical cord mesenchymal stem cells secrete peroxiredoxin 6 (PRDX6), a core immunoregulatory molecule, to reprogram macrophages toward a reparative phenotype and thereby alleviate the inflammatory response both locally and systemically [77]; and bone marrow mesenchymal stem cell–derived exosomes enriched in miR‐181a‐5p promote M2 polarization through ZEB2‐mediated ubiquitination and degradation of receptor for activated C kinase 1 (RACK1) while directly suppressing acinar cell apoptosis, thus achieving dual intervention against inflammation and tissue injury [78].

Despite their broad promise, such biologically sourced carriers still face common bottlenecks: Batch‐to‐batch variation in the protein composition of native cell membranes may compromise targeting stability, whereas exosomes typically suffer from low yield, complex purification, and marked batch‐to‐batch variability in cargo—challenges that warrant caution in both scale‐up production and clinical translation.

#### 3.3.2. Synthetic Biomimetic Nanocatalytic Platforms: On‐Demand Response to the Inflammatory Microenvironment

Unlike biomimetic carriers that rely on natural components, this class of materials functions primarily through its intrinsic catalytic activity, antioxidant capacity, or ion‐regulating properties and is characteristically activated in response to the inflammatory microenvironment (acidic pH and high ROS). This on‐demand activation mechanism itself endows it with spatiotemporal specificity.

Nanozymes can mimic the function of natural antioxidant enzymes. The Prussian blue nanozyme composite Cri‐Pt‐CaFePB integrates ROS scavenging with peroxidase‐like activity, driving macrophages toward a reparative phenotype, markedly reducing inflammatory cytokine levels, and improving histopathological scores in a SAP model [79]. The tungsten‐based heteropolyacid nano‐antioxidant mTWNDs further exemplify the refinement of spatial targeting: Its high affinity for type III collagen enables it to cross the damaged blood–pancreas barrier and accumulate in pancreatic tissue, where it scavenges mitochondrial ROS in acinar cells while simultaneously blocking the cGAS‐STING signaling pathway in macrophages—thereby suppressing inflammation at both the acinar and macrophage levels—at an effective dose only 1/50 that of N‐acetylcysteine [80].

Nanoscale delivery of effector molecules further enriches this category: bilirubin nanoparticles (BRSNPs) achieve targeted enrichment of bilirubin at inflammatory lesions and undergo enzyme‐responsive release, thereby coordinately modulating the NF‐κB and NRF2/HO‐1 pathways [81]. Notably, the regulatory targets of these designs are no longer confined to macrophages but extend to neutrophils and even to calcium signaling in acinar cells, suggesting that the key advantage of functional nanomaterials lies in their capacity to simultaneously intervene at multiple cellular nodes within the inflammatory network.

However, the long‐term in vivo accumulation and metabolic fate of most such materials remain unclear, and the preparation and large‐scale production of nanomaterials continue to pose practical obstacles to clinical translation.

#### 3.3.3. Nanoformulations of Bioactive Natural Products: A Route to Spatiotemporally Precise Delivery

The multitarget, multipathway nature of bioactive natural products endows them with unique potential for modulating macrophage function (see Section 3.2). However, inherent drawbacks—including poor water solubility, rapid metabolism, low bioavailability, and insufficient targeting—hinder their ability to achieve precise modulation at a specific phase and site. Nanotechnology, through rational carrier design, can simultaneously confer spatial targeting and temporally controlled release, offering a means to overcome these limitations.

Along the spatial dimension, the most straightforward strategy is to distinguish macrophage subsets through receptor recognition: Mannose‐modified emodin nanocapsules target the M2 subset via CD206, remodeling lipid metabolism to promote M1‐to‐M2 polarization in a model of SAP [82]; heparin‐modified carriers, in turn, restore macrophage mitochondrial function through CD44 targeting [83]. This subset specificity is, however, relative: CD206 and CD44 are not strictly exclusive markers of any single subset, and because the macrophage phenotype undergoes continuous dynamic transition over the disease course, relying on a receptor profile captured at a single time point can hardly enable real‐time, precise identification of subsets in flux.

An alternative route to spatial enrichment is biomimetic membrane coating. Macrophage membrane–coated nanoparticles codeliver proanthocyanidins and pentoxifylline to suppress the pathological recruitment of macrophages and neutrophils [84]. Selenized Poria cocos polysaccharide nanoparticles provide a complementary illustration: Selenization itself enhances anti‐inflammatory and autophagy‐modulating capacity but does not confer spatiotemporal selectivity; only by further coating the particles with a macrophage membrane—leveraging biomimicry to emulate the active homing of immune cells to inflammatory sites—can they evade clearance by the mononuclear phagocyte system, achieving enrichment and durable retention of the drug in the inflamed pancreatic tissue. This demonstrates that molecular optimization must be coupled with a targeted delivery vehicle for the full therapeutic benefit to be realized. Yet, membrane‐mediated homologous targeting generally achieves only tissue‐level enrichment and cannot dictate whether the cargo acts locally on proinflammatory or reparative subsets [85, 99].

The temporal dimension is most effectively harnessed by microenvironment‐responsive release. Early AP is characterized by acidic pH and high levels of ROS. One illustrative system employs Prussian blue to continuously scavenge ROS, while ZIF‐8 degrades in the acidic microenvironment to release celastrol and induce autophagy; coated with a macrophage membrane, this system raises survival from 12.5% to 62.5% in a lethal SAP model. Its value resides in the fact that drug release is triggered by the microenvironment rather than occurring through passive diffusion [86]. At present, however, such responsiveness remains unidirectional and single‐threshold—it cannot adaptively scale back as inflammation resolves nor can it accommodate the interindividual heterogeneity and temporal dysregulation of the microenvironment encountered in clinical AP.

From biomimetic carriers and synthetic nanozymes to natural‐product nanoformulations, these three classes of strategies collectively reflect a substantive shift in delivery design from passive cargo delivery to active responsiveness. Spatial precision, however, often remains at the tissue or receptor level and fails to capture subsets undergoing dynamic phenotypic transitions. Temporal responsiveness is likewise mostly designed as a unidirectional, single‐threshold trigger, making it difficult to achieve bidirectional modulation as inflammation resolves. Even setting aside engineering bottlenecks such as batch‐to‐batch stability, in vivo accumulation, and scale‐up production, the deeper predicament is that the spatiotemporal heterogeneity of macrophages has yet to be genuinely incorporated into the design language of delivery systems.

## 4. Challenges and Opportunities for Clinical Translation

Although preclinical mechanistic studies on macrophage recruitment, polarization, metabolism, and death are now abundant, no macrophage‐targeted therapy has yet entered clinical use for AP. Current delivery systems still address the spatiotemporal heterogeneity of macrophages in a piecemeal manner—typically by targeting a single subset marker, responding to a single pathological signal, or intervening at a single polarization node—without achieving systematic adaptation to the entire dynamic process. Underlying this piecemeal approach are three interrelated challenges: mechanistic phase reversal, intervention timing, and translational engineering.

First, the phase‐dependent reversal of therapeutic effects deprives intervention timing of an objective yardstick. The same target can exhibit diametrically opposite effects at different stages of disease: Inhibiting PFKFB3 attenuates injury in the inflammatory phase but delays tissue regeneration in the repair phase by cutting off lactate‐driven repair signals; MET‐mediated IL‐33 release contributes to proinflammatory amplification early on, yet in the mid‐to‐late phase, it facilitates the immune transition from SIRS to CARS. At present, there is neither a clear boundary demarcating the proinflammatory from the repair phase nor a biomarker capable of monitoring this transition in real time. Consequently, the most critical decision—when to switch from inhibition to promotion or even to actively drive repair—lacks an objective basis. This is precisely the major limitation that prevents phase‐specific interventions from reaching the clinic.

Second, the dual constraints of patient presentation time and drug therapeutic window further narrow strategic options. AP patients have often already passed the early 0–24 h window by the time they present, leaving a very narrow real‐world window for strategies aimed at blocking pathological monocyte recruitment. In a phase II trial, the platelet‐activating factor (PAF) antagonist lexipafant was shown to reduce inflammatory mediators. However, in a subsequent large confirmatory trial enrolling 290 patients with APACHE II scores > 6, 44% of patients already had organ failure at enrollment, while newly developed organ failure after enrollment accounted for only 14%. Even with the dosing window extended to 72 h, the systemic inflammatory burst had already become uncontrollable. This case clearly illustrates that once the early window is missed, upstream blocking strategies are all but destined to fail, and the therapeutic focus is therefore more likely to shift toward repair modulation and complication prevention in the mid‐to‐late phases. A more realistic point of entry is to rely on circulating biomarkers for patient stratification and phase determination. MIF, S100A9, and others have been associated with SAP and could potentially serve both as a stratification basis and as objective indicators for intervention timing. However, these biomarkers have been identified primarily in retrospective or single‐center studies, and whether they can truly guide the timing of intervention remains to be confirmed in prospective investigations.

Finally, safety concerns, off‐target effects, and translational engineering are obstacles that cannot be circumvented. Most key targets (MIF, Grb2, CD36, NAMPT, S1PR2, and others) are broadly expressed across various immune and nonimmune cells, making systemic intervention inherently prone to off‐target effects. Systemic inhibition of these molecules would impair anti‐infective defenses during the phase of infected necrosis—which is precisely the leading cause of death in late‐phase SAP. Although nanoscale drug delivery offers the potential for spatial targeting and temporally controlled release, it also introduces new translational hurdles: The batch‐to‐batch variability in the protein composition of biomimetic membranes undermines targeting consistency; the long‐term in vivo accumulation and metabolic fate of most nanomaterials remain unclear; and the reproducible preparation, scale‐up manufacturing, and regulatory pathways for composite carriers still lack well‐established protocols. Moreover, the available evidence derives almost entirely from rodent models, whereas human AP is highly heterogeneous in etiology, disease course, and immune background. Given the interspecies differences and the paucity of clinical validation data, any extrapolation of current findings to the clinical setting must be undertaken with extreme caution.

It is thus evident that the key to clinical translation of macrophage‐targeted therapy lies no longer in identifying new molecular nodes but in coordinated advances on three fronts: phase‐discriminating biomarkers to objectively define the timing of intervention; delivery systems selective for specific subsets and microregions to mitigate off‐target effects and immune‐related toxicity; and the establishment of models that more closely recapitulate human pathology, upon which early‐stage clinical trials stratified by disease‐phase biomarkers can be designed. Only in this way can macrophage‐targeted therapy progress from a mechanistic concept to clinical reality.

## 5. Conclusions and Perspectives

The function of macrophages in AP is not a simple dichotomy of proinflammatory versus anti‐inflammatory but rather a dynamic, continuous spectrum jointly governed by cellular origin, disease phase, and the local microenvironment. Centered on spatiotemporal heterogeneity, this review has delineated the spatial division of labor and the shift in dominance between TRMs and MDMs, as well as the temporal progression from early proinflammatory initiation, through an intermediate phase in which injury and repair coexist, to a late repair‐dominated phase. By incorporating single‐cell and spatial multiomics evidence, we have discussed functional subsets and spatial niches that extend beyond the M1/M2 framework. These insights have enabled macrophage‐targeted strategies to move from broad‐spectrum anti‐inflammatory intervention toward precise modulation directed at specific phases, specific subsets, and specific regions. Yet, as discussed above, the response to spatiotemporal heterogeneity remains piecemeal—the bottleneck no longer lies in identifying new molecular nodes but in delivering an intervention to the right cell population, at the right time, and in the right place, as it undergoes continuous phenotypic transition.

Building on this analysis, we contend that dual‐dimensional (spatiotemporal) precision modulation represents a priority for validation and outline testable research paths. In the spatial dimension, single‐cell and spatial transcriptomics can be leveraged to identify macrophage subsets such as CCL3^+^, PF4^+^PPBP^+^, SPP1^+^, and NLRP3^+^ and to evaluate their dual utility as markers for patient stratification and as targets for drug delivery. The next generation of nanocarrier‐based delivery systems should also evolve from targeting a single subset marker toward continuously tracking and selectively targeting functional subsets during dynamic phenotypic succession. In the temporal dimension, beyond disease staging, circadian rhythmicity is an underexplored variable. Macrophage polarization exhibits diurnal oscillations regulated by the core clock genes Bmal1 and Clock [100, 101]. Therefore, the rhythmic profiles of iNOS, Arg‐1, and CD206 could be characterized in standard AP models, and controlled experiments with equivalent dosing at different circadian time points could be designed to ascertain the specific contribution of timing. Furthermore, employing Bmal1 conditional knockout models would allow dissection of the reciprocal regulation between the circadian clock and disease progression on macrophage polarization programs. For therapeutic exploration, a temporally sequenced combination regimen could be devised: administering proinflammatory inhibitors such as baicalin and paeonol during the peak of acute inflammation and repair promoters such as emodin and tectoridin during the recovery phase, with nontemporally sequenced dosing serving as a control to evaluate the advantages of this approach. This concept aligns with the traditional Chinese medical principle of *yin shi zhi yi* (adapting treatment to timing), the core of which is to restore the disordered immune rhythm to its normal phase.

Nevertheless, the hypotheses outlined above are currently based largely on rodent evidence. The pronounced heterogeneity of human AP presents persistent species‐translation challenges, and the majority of these strategies remain at the preclinical stage. The value of these hypotheses lies in providing experimentally testable designs that, if validated step by step, could help propel AP therapy from symptomatic support toward cause‐directed repair. Achieving this goal will require interdisciplinary collaboration across immunology, chronobiology, and integrative traditional Chinese and Western medicine.

**Nomenclature**
AP: Acute pancreatitis
TRMs: Tissue‐resident macrophages
MDMs: Monocyte‐derived macrophages
SAP: Severe acute pancreatitis
SIRS: Systemic inflammatory response syndrome
MODS: Multiple organ dysfunction syndrome
CCL2: C–C motif chemokine ligand 2
CCR2: C chemokine receptor type 2
Arg1: Arginase 1
ECM: Extracellular matrix
ADM: Acinar‐to‐ductal metaplasia
NLRP3: NOD‐like receptor family pyrin domain containing 3
HMGB1: High‐mobility group box 1
DAMPs: Damage‐associated molecular patterns
scRNA‐seq: Single‐cell RNA sequencing
IL‐10: Interleukin‐10
TGF‐β: Transforming growth factor beta
NF‐κB: Nuclear factor kappa B
MLKL: Mixed‐lineage kinase domain‐like protein
RIPK3: Receptor‐interacting protein kinase 3
CSF1R: Colony stimulating factor 1 receptor
HGF: Hepatocyte growth factor
EGF: Epidermal growth factor
PSCs: Pancreatic stellate cells
PDGFRα^+^: Platelet‐derived growth factor receptor alpha
MIF: Macrophage migration inhibitory factor
Grb2: Growth factor receptor‐bound protein 2
PFKFB3: 6‐Phosphofructo‐2‐kinase/fructose‐2,6‐bisphosphatase 3
NRF2: Nuclear factor erythroid 2–related factor 2
HO‐1: Heme oxygenase 1
FAEE: Fatty acid ethyl ester
NAMPT: Nicotinamide phosphoribosyltransferase
NMN: Nicotinamide mononucleotide
ATMs: Adipose‐tissue macrophages
S1PR2: Sphingosine‐1‐phosphate receptor 2
METs: Macrophage extracellular traps
CARS: Compensatory anti‐inflammatory response syndrome
ROS: Reactive oxygen species
PAD: Peptidylarginine deiminase
GDF11: Growth differentiation factor 11
PPARγ: Peroxisome proliferator–activated receptor γ
p‐MLKL: Phosphorylated mixed–lineage kinase domain‐like protein
AKBA: Acetyl‐11‐keto‐β‐boswellic acid
ERK2: Extracellular signal–regulated kinase 2
AP‐ALI: AP‐associated acute lung injury
SCFAs: Short‐chain fatty acids
HDACs: Histone deacetylases
SSTR2: Somatostatin receptor 2
EVs: Extracellular vesicles
PRDX6: Peroxiredoxin 6
RACK1: Receptor for activated C kinase 1
BRSNPs: Bilirubin nanoparticles
PAF: Platelet‐activating factor.

## Author Contributions

Zhi Li, Xiao‐ning Jin, and Chao‐li Jiang designed the study and coordinated technical support and funding. Xin Zhou revised the manuscript. Xiang Lu and Yu Zeng performed the study, drafted the manuscript, and designed the figures. Li‐hong Gan conducted the literature search and screening. Li Li and Jian‐qin Liu participated in the study.

## Funding

This study was supported by the following grants: Science and Technology Project of Sichuan Administration of Traditional Chinese Medicine (Grant 2024zd035), Sichuan University and Luzhou Municipal People’s Government Strategic Cooperation Projects (Grant 2020CDLZ‐18), Southwest Medical University Technology Program (Grant 2023ZYQJ03) and Sichuan Provincial Administration of Traditional Chinese Medicine Special Scientific and Technological Research Project (Grant 2024MS020).

## Disclosure

All authors have read and approved the final manuscript.

## Conflicts of Interest

The authors declare no conflicts of interest.

## Data Availability

Data sharing is not applicable to this article as no datasets were generated or analyzed during the current study.
